# Anomalous Hall-like effect probe of antiferromagnetic domain wall

**DOI:** 10.1038/s41598-017-18514-4

**Published:** 2018-01-10

**Authors:** Lili Lang, Xuepeng Qiu, Shiming Zhou

**Affiliations:** Shanghai Key Laboratory of Special Artificial Microstructure Materials and Technology and Pohl Institute of Solid State Physics and School of Physics Science and Engineering, Tongji University, Shanghai, 200092 China

## Abstract

Of crucial importance to antiferromagnetic (AF) spintronic devices, AF domain wall (AFDW), created in exchange biased Y_3_Fe_5_O_12_/Ni_0.50_Co_0.50_O (NiCoO)/Pt, is characterized by anomalous Hall-like effect through magnetic proximity effect and spin Hall magnetoresistance at NiCoO/Pt interface. The AFDW thickness, in the order of nanometers, has been for the first time proved in experiments to increase with increasing temperature. AF spins within AFDW show the same chirality in decent and ascent branches of ferromagnetic magnetization reversal process. Moreover, the uncompensated magnetic moment at the NiCoO/Pt interface is of perpendicular magnetization anisotropy and changes linearly in magnitude with temperature due to the reduced coordination of the magnetic atoms on the AF surface. This work will help to clarify the mechanism of the spin current propagation in AF materials and fully understand the physics behind exchange bias.

## Introduction

Antiferromagnetic (AF) materials are becoming increasingly important in the newly merging AF spintronics field^1–3^. While they have been playing a central role in devices of spin valves and magnetic tunneling junction as the pinning layer^4^, AF materials are also found in enabling many other intriguing phenomena^5–13^. For example, tunneling anisotropy magnetoresistance, induced by AF anisotropic density of states near Fermi surface, has been found in AF-based tunnel junctions^8,9^. In particular, efficiency enhancement of spin pumping and thermal spin current injection has been observed after insertion of insulating AF layer between ferromagnet (FM) and normal metal (NM) layers^10–13^.

**Figure 1:**
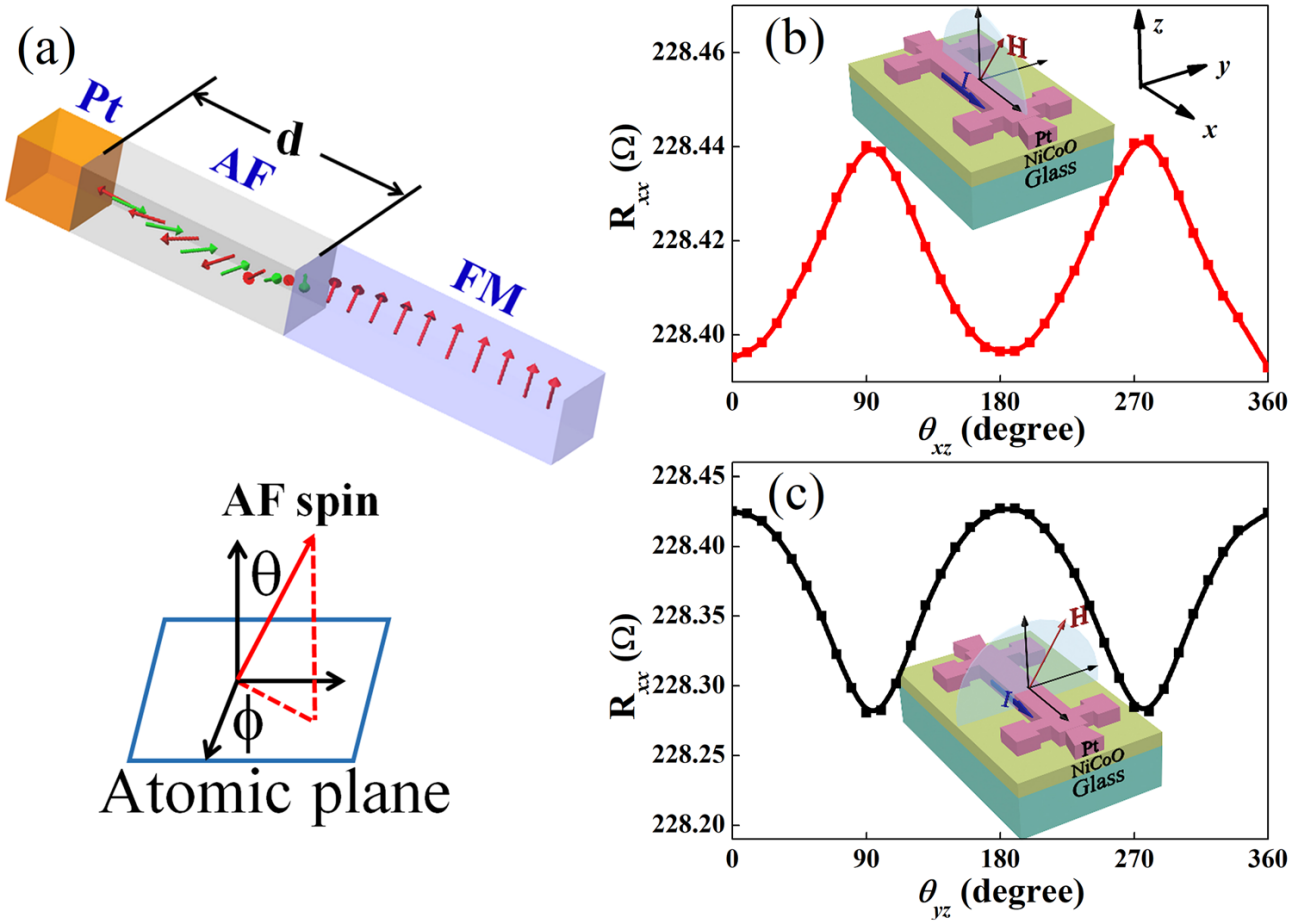
Schematic geometries of hybrid planar DW in YIG/NiCoO/Pt (**a**), of anisotropic magnetoresistance (**b**) and spin Hall magnetoresistance (**c**) measurements.

Recently, the pioneered theoretical works have proposed the new generation spintronic devices based on AF domain wall, i.e., AFDW^14–16^. Due to the negligible magnetic moment and the alternating exchange field of AF material, AFDW can be driven at an unprecedented velocity by spin orbit torques and spin waves. Thus, creating AFDW and revealing the characteristics of AFDW are of crucial importance for exotic AF spintronics devices. On the other hand, AF exchange correlation length and AFDW thickness have great impact on the spin pumping and the thermal spin current injection^10–13^. It is also believed that AFDW governs the phenomena of exchange bias^17–23^. In this work, we will study the AFDW thickness as a function of temperature. Here, hybrid domain wall is created by exchange bias in Y_3_F_5_O_12_ (YIG 10.0 nm)/NiCoO/Pt (5.0 nm) heterostructures^19^, as shown in Fig. 1a. The AF attribute of ultrathin NiCoO layers is confirmed by the exchange bias establishment in NiCoO/NiFe and YIG/NiCoO bilayers (Figs S1 and S2)^24^. For typical magnetically soft YIG layers epitaxially grown on Gd_3_Gd_5_O_12_ substrates, the FM domain wall thickness is about 100 nm due to small magnetic anisotropy^25^, much larger than the YIG thickness (10.0 nm), and thus the angular evolution of spin orientation in hybrid domain wall is mainly accomplished in the AF layer.

The motion of AF spins at NiCoO/Pt interface and thus the AFDW are characterized by anomalous Hall-like effect (AHLE) which in turn consists of anomalous Hall effect of polarized Pt atoms due to the magnetic proximity effect (MPE) of nearly ferromagnetic Pt, as shown in Fig. 1b and the uncompensated magnetic moment (UMM) induced spin Hall magnetoresistance at the Pt/NiCoO interface^26–30^, as shown in Fig. 1c. The spin Hall magnetoresistance, anisotropic magnetoresistance, and AHLE read^26,27,31^

1 $${\rho }_{xx}={\rho }_{0}+{\rm{\Delta }}{\rho }_{1}{m}_{y}^{2},$$

2 $${\rho }_{xx}={{\rm{\rho }}}_{0}+{\rm{\Delta }}{\rho }_{2}{m}_{x}^{2},$$

3 $${\rho }_{xy}={\rho }_{AHLE}{m}_{z},$$

where ${\rho }_{0}$ refers to the longitudinal resistivity at zero *H*, and ${m}_{x}$, ${m}_{y}$, ${m}_{z}$ are the projections of the UMM ${m}_{{UMM}}$ along *x*, *y*, and *z* axes, respectively, as shown in the inset of Fig. 1b and c. Moreover, AHLE at low magnetic fields may also be contributed by planar Hall effect (PHE) resistivity^32^, i.e., ${\rm{\Delta }}{\rho }_{2}{m}_{x}{m}_{y}$, in addition to contributions of spin Hall magnetoresistance and MPE^31^.

The shape of the Hall loop depends on the NiCoO layer thickness. When the NiCoO layer is thicker (thinner) than the correlation length, i.e. the AFDW thickness, the UMM and the YIG magnetization are decoupled (coupled), and thus the shape of the Pt Hall loop are independent (dependent) of the magnetization reversal process of the YIG layer. Therefore, the AFDW thickness can be measured as a function of temperature by the Hall loop of the Pt layer. With the present approach, the motion of the UMM at NiCoO/Pt interface is directly probed in experiments. In contrast, in the X-ray linear magnetic dichroism^18,19^, the optical signal is contributed by all atomic layers within the penetration depth region and the motion of individual atomic layer cannot be detected without numerical simulation.

## Results and Discussion

**Figure 2:**
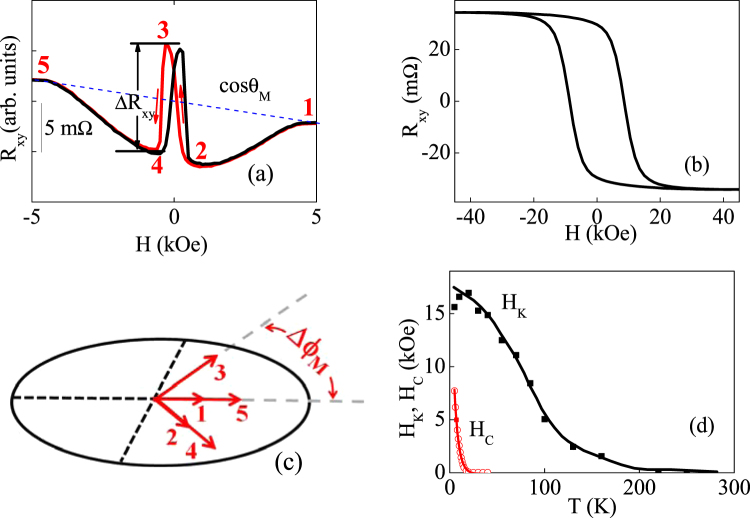
AHLE loops of YIG (10 nm)/NiCoO (1.25 nm)/Pt (5.0 nm) at 100 K (**a**) and NiCoO (2.0 nm)/Pt (5.0 nm) at 5 K (**b**). Schematic geometry (**c**) of evolution of the azimuthal orientation ${\varphi }_{M}$ of the UMM among consequent stages from stage 1 to stage 5 in (**a**). *T* dependencies of magnetic anisotropic field *H*_*K*_ and out-of-plane coercivity *H*_*C*_ at *θ*_*H*_ = 0 (**d**). In (**d**), black lines serve a guide to the eye.

The MPE helps to analyze the motion of the UMM at NiCoO/Pt interface during the FM magnetization reversal process. The butterfly-shaped AHLE loop for YIG/NiCoO/Pt in Fig. 2a is distinguished from that of NiCoO/Pt in Fig. 2b. When the UMM and thus the Pt induced magnetic moment are deviated from *z* axis at low *H*, their azimuthal angles change with *H* due to the in-plane anisotropy of the epitaxial YIG layers. Accordingly, PHE of polarized Pt atoms is also included in the transverse resistivity ${\rho }_{{xy}}$ ^31,32^, and the latter one obeys the equation,$\,{\rho }_{xy}={\rho }_{AHLE}\,\cos \,{\theta }_{M}+{\rm{\Delta }}{\rho }_{2}{\sin }^{2}{\theta }_{M}\,\sin (2{\rm{\Delta }}{\varphi }_{M})$. Therefore, *R*_*xy*_ at the stage 3(2, 4) is larger (smaller) than that of the negative (positive) saturation. From stage 1 to stage 5 in the decent branch, i.e., from the positive to the negative *H*, the azimuthal angle ${\varphi }_{M}$ evolves in a way in Fig. 2c. Meanwhile, the polar angle ${\theta }_{M}$ changes from 0 to 180 degrees through 90 degrees from stage 1 to stage 5. Moreover, the hysteresis behavior of the UMM is observed between decent and ascent branches. The reversal process of the UMM in two branches is symmetric, exhibiting the same chirality. The mechanism of the AFDW chirality may depend on the interfacial Dzyaloshinskii–Moriya interaction (DMI) at the YIG/NiCoO and NiCoO/Pt interfaces^33–36^. The coexistence of AFDW and Dzyaloshinskii–Moriya interactions enable novel antiferromagnetic spintronic applications such as spin wave polarizer and retarder^37^. Moreover, it is suggested the Dzyaloshinskii–Moriya interactions in antiferromagnets can lead to more robust antiferromagnetic Skyrmions compared to its ferromagnetic counterpart due to the insensitivity to stray field and cancellation of Magnus force^38,39^. It is noted that although the *R*_*xy*_ change from stage 2(3) to 3(4) is mainly contributed by the PHE, other contributors of AHLE cannot be excluded.

The squared AHLE loop in NiCoO (2.0 nm)/Pt (5.0 nm) after field cooling procedure indicates the UMM and the MPE at NiCoO/Pt interface and the establishment of perpendicular magnetic anisotropy in both the UMM and the induced Pt atomic moment, as shown in Fig. 2b, because ${\rho }_{xy}$ is contributed by the AHLE, without any contribution from PHE^31^. The anisotropic magnetic field *H*_*K*_ is deduced from the measured angular dependence of spin Hall magnetoresistance in the *yz* plane (Fig. S3)^24^, and it is as large as 16.0 kOe at 5 K and decreases with increasing *T*, as shown in Fig. 2d. For the AF layer of the uniaxial anisotropy, the anisotropic magnetic field $\,{H}_{K}=2{K}_{U}/{m}_{UMM}$, where $\,{K}_{U}\propto {M}_{AF}^{n(n+1)/2}$ with n = 2 and ${M}_{AF}$ being the magnetization of the AF sublattice^40,41^. Since the UMM also decreases with increasing *T*, as discussed below, ${M}_{AF}$ decreases with increasing temperature. Meanwhile, the out-of-plane coercivity *H*_C_ decreases with increasing *T*. When the angle between *H* and the film normal direction, ${\theta }_{H}$, increases, the angular dependence deviates from the scaling law of $1/\,\cos \,{\theta }_{H}$ and the magnetization reversal process is accompanied by modified Kondorsky model^42^, as shown in supplementary information (Fig. S4)^24^.

**Figure 3:**
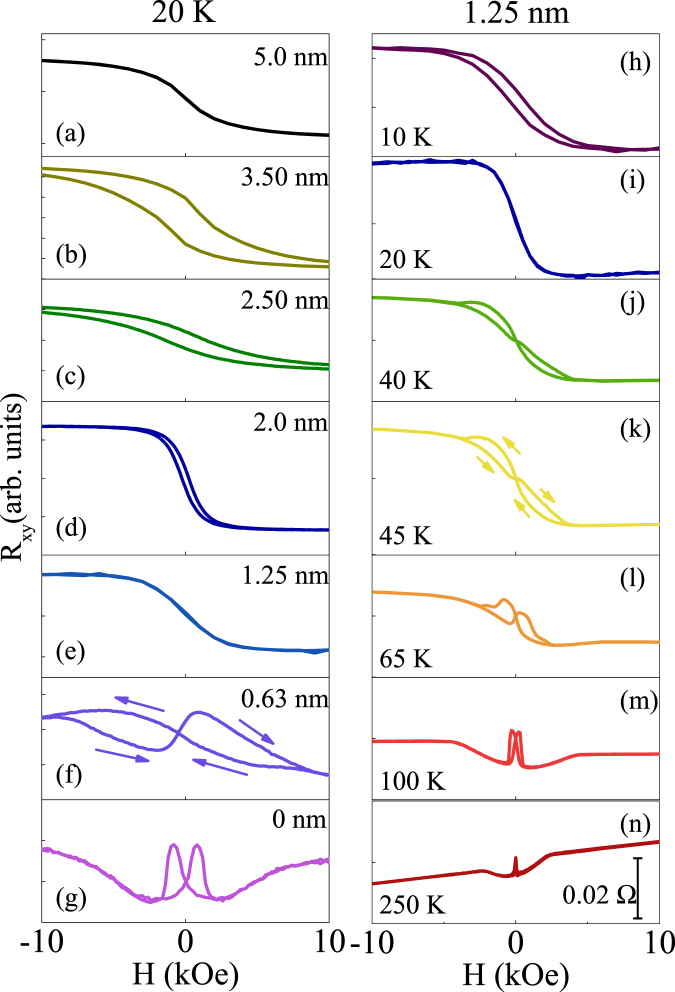
At 20 K, AHLE loops for YIG (10 nm)/NiCoO (d)/Pt (5.0 nm) with *d* = 5.0 (**a**), 3.50 (**b**), 2.50 (**c**), 2.0 (**d**), 1.25 (**e**), 0.63 (**f**), 0 (**g**) (nm) in the left column. AHLE loops for YIG (10 nm)/NiCoO (d)/Pt (5.0 nm) with 1.25 nm thick NiCoO layers at different temperatures (**h**–**n**) in the right column.

**Figure 4:**
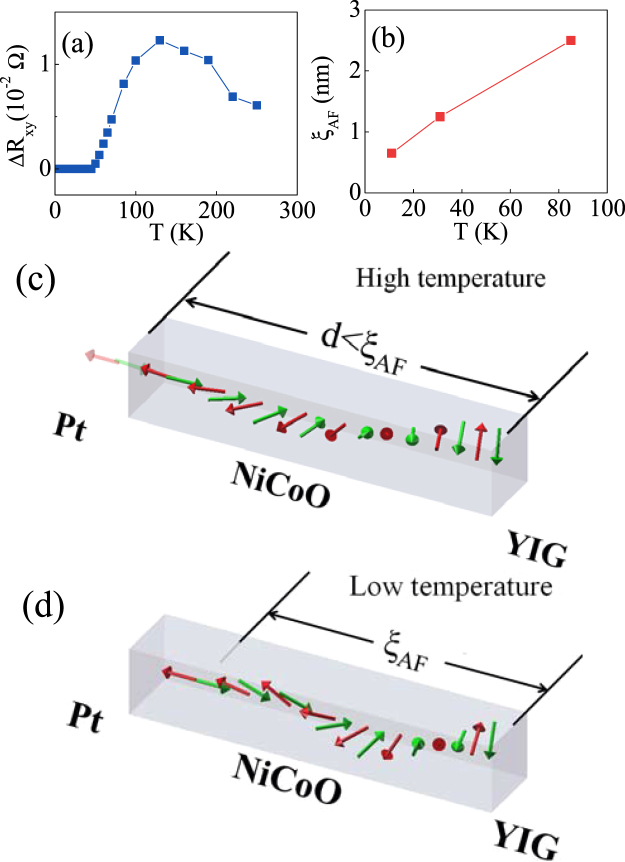
(**a**) Δ*R*_*xy*_ versus *T* for *d* = 1.25 nm and (**b**) AFDW thickness versus temperature for YIG/NiCoO/Pt. At low *H*, partial AFDW at high *T* (**c**) and full one at low *T* (**d**). Here, Δ*R*_*xy*_ is defined in Fig. 2a.

The establishment of antiferromagnetic order of NiCoO, as well as the exchange coupling between YIG and NiCoO have been confirmed by the magnetization hysteresis loop measurements on NiCoO/NiFe and YIG/NiCoO samples (Figs S1 and S2)^24^. While the UMM at NiCoO/Pt interface orients perpendicularly (Fig. 2b), the AF spins at YIG/NiCoO is pinned in-plane by the interfacial interlayer coupling between YIG and NiCoO. Thus, an atomic scale AFDW, as schematically shown in Fig. 1a, is formed inside the NiCoO layer. Figs 3 and 4 show the transport measurement results of AFDW. The AHLE loops in the right column of Fig. 3 and the results in Fig. 4a show that ${\rm{\Delta }}{R}_{xy}$, as defined in Fig. 2a, changes nonmonotonically with *T*, exhibiting the maximum at an intermediate temperature. Similar results are also observed for other NiCoO thickness *d* values, as shown in supplementary information (Fig. S5)^24^. Here, ${\rm{\Delta }}{R}_{xy}$ is proportional to both ${\rm{\Delta }}{\rho }_{2}$ and $\sin (2{\rm{\Delta }}{\varphi }_{M})$. ${\rm{\Delta }}{\rho }_{2}\,\,$decreases monotonically with increasing *T*, as shown below. Meanwhile, ${\rm{\Delta }}{\varphi }_{M}$ for a fixed AF layer thickness *d*, as defined in Fig. 2c, increases monotonically when AFDW thickness *ξ*_*AF*_ increases with increasing *T*. ${\rm{\Delta }}{\varphi }_{M}$ approaches the saturated value, i.e., that of the FM magnetization when $d\ll {\xi }_{AF}$ at high temperatures and it equals zero for $d\gg {\xi }_{AF}$ at low temperatures, as shown in Fig. 4c,d, the UMM is therefore coupled (decoupled) with the FM magnetization at high (low) temperatures^22,43,44^. It is further confirmed by the results in the left column of Fig. 3 that AHLE loops of YIG/NiCoO/Pt can be classified into two types. For *d*=0.63 nm, the loop of YIG/NiCoO/Pt is similar to that of Pt/YIG in Fig. 3g and the reversal process of the UMM is modified by the FM magnetization. In contrast, AHLE loops in Fig. 3a–e for thick NiCoO layers, are similar to that of NiCoO/Pt in Fig. 2b and the reversal of the UMM is independent of the FM magnetization and directly controlled by *H*. Equivalently, the nonmonotonic variation of ${\rm{\Delta }}{R}_{xy}$ favors to map the evolution of the azimuthal angle of AF spins with the atomic plane index during the FM magnetization reversal process, as shown in Fig. 1a.

Since the UMM has perpendicular magnetic anisotropy and negligible in-plane anisotropy due to its polycrystalline structure (Fig. S6)^24^ whereas the epitaxial YIG layer, as an in-plane film, has an in-plane magnetic anisotropy, *both* polar and azimuthal angles of AF and FM spins change with the atomic plane index during the FM magnetization reversal process. Exchange spring may occur in both FM and AF layers, i.e., forming planar hybrid DW, or in either AF or FM layer^17,19^. The evolutions of polar and azimuthal angles of AF spins, as shown in Fig. 1a, arise from the competition of the demagnetization energy of the FM layer and the perpendicular magnetic anisotropy of the AF layer, and from the magnetic anisotropy of the epitaxial YIG layer, respectively. The present AF/FM heterostructures with perpendicularly magnetized AF and in-plane magnetized FM exhibit a new type of DW, different from Bloch and Néel DWs in pure FMs in which either the polar angle or the azimuth one changes with the atomic plane index.

It is significant to investigate the AFDW thickness as a function of temperature. Continuous tuning of temperature allows for precise measurements of *ξ*_*AF*_ as a function of *T*. AHLE loops in Fig. 3h,i are close to that of NiCoO/Pt, that is to say, ${\rm{\Delta }}{R}_{xy}=\,0$ at *T* < 40 K and the onset temperature for nonzero ${\rm{\Delta }}{R}_{xy}$ is about 40 K for *d* = 1.25 nm, as shown in Fig. 4a. The onset temperature is about 11 K and 85 K for *d* = 0.63 and 2.5 nm (Fig. S7), respectively^24^. It can be further confirmed by the results in the left column of Fig. 3, 0.63 < *ξ*_*AF*_ < 1.25 nm at 20 K and 1.25 < *ξ*_*AF*_ < 2.5 nm at 65 K (Fig. S8)^24^. Apparently, the AFDW thickness has been for the first time proved *in experiments* to increase with increasing *T*, as shown in Fig. 4b. For the present uniaxial magnetic anisotropy^40,41^, ${K}_{U}\propto {M}_{AF}^{3}$, and the exchange stiffness ${A}_{AF}\propto {M}_{AF}^{2}$ in analogy to FMs^45^, and therefore ${\xi }_{AF}\propto \sqrt{{A}_{AF}/{K}_{U}}\propto \sqrt{1/{M}_{AF}}$. When the AF sublattice magnetization ${M}_{AF}$ decreases with increasing *T*, as discussed below, the AFDW becomes thick at high temperatures, as shown in Fig. 4b.

**Figure 5:**
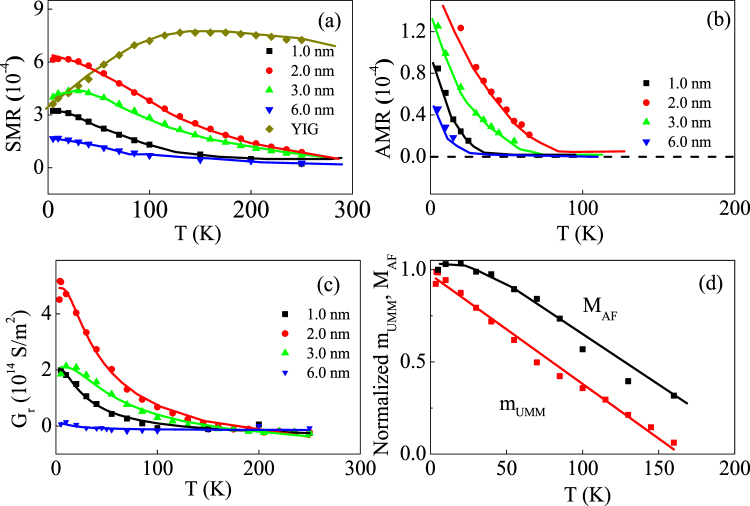
For NiCoO/Pt heterostructures, spin Hall magnetoresistance (**a**), anisotropic magnetoresistance (**b**), and *G*_*r*_ (**c**), and normalized *M*_*AF*_ and ${m}_{{\rm{UMM}}}$ (**d**) versus *T*. In (**a**), the data of YIG (80.0 nm)/Pt (4.0 nm) are given for comparison. The inset numbers (**a**,**b**,**c**) refer to the *d* value. The lines serve a guide to the eye.

Spin Hall magnetoresistance results in Fig. 5a can be employed to address the temperature dependence of the UMM. In comparison, anisotropic magnetoresistance results are also given in Fig. 5b. For all NiCoO/Pt samples, anisotropic magnetoresistance and spin Hall magnetoresistance both decrease with increasing *T*. In particular, spin Hall magnetoresistance in Fig. 5a shows different *T* dependencies in NiCoO/Pt and YIG/Pt^31^. The spin Hall magnetoresistance ratio reads^26,27^

4 $${\rm{\Delta }}{\rho }_{1}/{\rho }_{0}={\theta }_{SH}^{2}\frac{{\lambda }_{sd}}{{d}_{NM}}\frac{{\tanh }^{2}({d}_{NM}/2{\lambda }_{sd})}{1/2{\rho }_{0}{\lambda }_{sd}{G}_{r}+\,\coth ({d}_{NM}/{\lambda }_{sd})},$$

with spin diffusion length ${\lambda }_{sd}$, spin Hall angle $\,{\theta }_{SH}$, spin mixing conductance ${G}_{r}$, and the NM layer thickness ${d}_{NM}$. Since spin diffusion length and spin Hall angle are determined by physics properties of Pt layers, it is suggested that Pt layers in both NiCoO/Pt and YIG/Pt systems have close ${\lambda }_{sd}$ and ${\theta }_{{SH}}$. Accordingly, the large difference in the spin Hall magnetoresistance between two systems is mainly caused by the spin mixing conductance. After considering the constant spin mixing conductance of YIG/Pt below room temperature^46^, the spin mixing conductance of NiCoO/Pt is deduced to change sharply with temperature, as shown in Fig. 5c.

As discussed below, the strong T dependence of spin mixing conductance at NiCoO/Pt interface arises from sharp variation of the UMM. The correlation between spin mixing conductance and FM magnetization has been observed in FM/NM as a function of *T* ^46^. The spin mixing conductance changes little below room temperature when the FM magnetization is almost constant below 300 K for FM=Fe, Co, NiFe alloys, and etc^46^. With reasonably high Curie temperature of 580 K, the YIG magnetization is reduced by 10% with *T* from 5 K to 300 K. In contrast, the phase transition temperature of ultrathin AF layers is below room temperature and thus the UMM should change sharply for *T* < 300 K, leading to the strong *T* dependence of the spin mixing conductance. According to the model proposed by Ohnuma *et al*.^10,47^, the spin mixing conductance $${G}_{r}$$ at insulating-AF/NM interface is proportional to the *sd* interaction energy $${J}_{sd}^{2}$$ between *s* electrons in the NM layer and the AF atomic magnetic moment and to $$ < {S}_{AF0}{ > }^{2}$$ with $$ < {S}_{AF0} > $$ being the spins on the AF surface. Since the UMM on the AF surface $${m}_{UMM}\propto  < {S}_{AF0} > $$, the temperature dependence of $${m}_{UMM}$$ is achieved, as shown in Fig. 5d. In comparison, Fig. 5d also shows the *T* dependence of $${M}_{AF}$$, which is achieved from the results of *H*_*K*_ in Fig. 2d. Interestingly, the UMM changes more sharply than $${M}_{{\rm{AF}}}$$ because of reduced coordination and exchange interaction energy of the AF surface spins^48^.

The present work helps to unravel characteristics of the exchange bias. The hysteresis behaviors of spin Hall magnetoresistance and anisotropic magnetoresistance angular dependencies under high *H* (Fig. S3)^24^ and the strong perpendicular magnetic anisotropy directly elucidate the rotational hysteresis loss and the hysteretic behavior of angular dependence of exchange bias systems^4,49,50^. The observed perpendicular magnetic anisotropy in the AF layer also explains the perpendicular exchange bias in CoO/NiFe and CoO/[Co/Pt]_*n*_ multilayers^51,52^. The decrease of the AFDW energy at high *T* can well explain the *T* variation trend of the exchange bias^4^. Moreover, the AHLE loop for *d* = 1.25 nm is asymmetric near 40 K and symmetric at higher temperatures, as shown in the right column of Fig. 3. This phenomenon reproduces one of the fingerprints for the exchange bias, i.e., asymmetric/symmetric FM magnetization reversal process at low and high temperatures^4^. Finally, the Mauri model is proved in the present YIG/NiCoO systems^17^.

The results presented in this work are helpful to understand spin current transport. With weak magnetic anisotropy, the YIG domain wall thickness is expected to be about 100 nm^53^, much larger than the FM thickness of 10 nm, and the minor (major) angular evolution of the hybrid domain wall in YIG/NiCoO is therefore accomplished in the FM (AF) layer. The AFDW thickness in YIG/NiCoO/Pt is close to the exchange correlation length in the NiCoO layers, and the latter one is in turn proved to be in the same order as those of CoO and NiO^54,55^. The phenomena are well explained that only in the case of ultrathin AF layers occurs the efficiency enhancement of thermal spin current and spin pumping in the FM/AF/NM sandwiches^10,11,56^. For the AF thickness *d* < *ξ*_*AF*_, magnons propagate from the bottom FM/AF interface to the upper AF/NM one in experiments of spin pumping and thermal spin current injection in FM/AF/NM^10,11,56^, and the transmission efficiency of spin current achieves the maximum near the critical temperature of the AF layer. For *d* > *ξ*_*AF*_, less magnons reach at the upper interface and the efficiency of spin pumping and thermal spin current injection is reduced.

## Conclusion

In summary, the AFDW is created in exchange biased YIG/NiCoO/Pt heterostructures and its thickness is characterized by the AHLE to increase with increasing *T*. For *d* < (>)*ξ*_*AF*_, motions of the UMM and the FM magnetization are coupled (decoupled) to each other. More remarkably, the present perpendicularly magnetized-AF/in-plane-FM heterostructures exhibit a new type of two-fold spiral DW, in which polar and azimuthal angles change with the atomic plane index. AF spins show the same switching chirality in decent and ascent branches of the FM magnetization reversal process. Moreover, the UMM at AF/NM interface changes more sharply with temperature due to the reduced coordination of AF surface atoms, compared with the AF sublattice magnetization ${M}_{{\rm{AF}}}$. The present work will facilitate to understand spin current transport in AF spintronics devices and the exchange bias phenomena. Finally, the perpendicular magnetic anisotropy-AF/heavy-NM is of great potential in spin orbit torque devices.

## Experimental

A series of YIG (10.0 nm)/NiCoO/Pt (5.0 nm) and YIG (10.0 nm)/Pt (5.0 nm) heterostructures were prepared on Gd_3_Ga_5_O_12_ single crystals. In comparison, NiCoO/Pt (5.0 nm) and NiCoO/NiFe (10.0 nm) were deposited on glass substrates. The YIG layers were grown on (111)-oriented single crystalline Gd_3_Ga_5_O_12_ substrates by pulsed laser deposition (PLD). The base pressure of the PLD system is better than 2.0×10^−6^ Pa. NiCoO layers were fabricated by ac magnetron sputtering, and Pt and NiFe layers were prepared by dc magnetron sputtering at ambient temperature. The base pressure of the sputtering system is 5.0×10^−6^ Pa. The deposition rates of NiCoO, Pt and NiFe films were about 0.013, 0.126 and 0.042 (nm/s), respectively.

## Electronic supplementary material

Supplementary information
