# Supplementary material for: Anomalous Hall-like effect probe of antiferromagnetic domain wall
Source: Sci Rep. 2018 Jan 10;8:329. doi: 10.1038/s41598-017-18514-4 (PMC5762948; doi:10.1038/s41598-017-18514-4)
Supplement: Supplementary file 1 — Supplementary information [file 41598_2017_18514_MOESM1_ESM.pdf]

Supplementary information

Anomalous Hall-like effect probe of antiferromagnetic domain wall

Lili Lang, Xuepeng Qiu, and Shiming Zhou**

Exchange bias in NiCoO/NiFe and YIG/NiCoO

Figure S1a shows magnetization hysteresis loops of NiCoO (2.0 nm)/NiFe (10.0 nm) bilayer at different temperatures, after the sample was cooled from 385 K to 5 K in an in-plane magnetic field of 20 kOe. Clearly, the hysteresis loop is shifted to the negative magnetic field and the exchange bias (EB) is established. Figure S1b-S1c show the exchange field H_E as a function of sampling temperature T and NiCoO layer thickness d. Figure S2a shows magnetization hysteresis loops of YIG (10.0 nm)/NiCoO (5.0 nm) bilayer at 55 K and 100 K after an in-plane cooling process. Figure S2b-S2c show the temperature dependence of H_E and H_C in YIG (10.0 nm)/NiCoO (5.0 nm), respectively.

Magnetic anisotropic field H_K and out-of-plane coercivity H_C

Typical angular dependent spin Hall magnetoresistance (SMR) results of NiCoO (2.0 nm)/Pt (5.0 nm) at an external magnetic field H=70 kOe and T=5 K, are shown in Figure S3a. The hysteretic behavior apparently exists between clock wise and counter clock wise rotations, indicating that the irreversible rotation of uncompensated magnetic moment (UMM) at NiCoO interface when H is aligned along film plane. The magnetic anisotropic field H_K is fitted from the angular dependent SMR results in Figure S3b, with the free energy for NiCoO/Pt heterostructures with perpendicular magnetic anisotropy, $E=\boldsymbol{M}_{\mathrm{UMM}}\times\boldsymbol{H}-2\pi M_{UMM}^2\cos^2\theta_M+K_u\cos^2\theta_M$ where M_{UMM} is the UMM on NiCoO surface, K_u is uniaxial anisotropy constant and θ_M (θ_H) is the polar angle between UMM (H) and the film normal direction z-axis.

When the angle between H and the film normal direction, θ_H, increases, the angular dependence of H_C deviates from the scaling law of $1/\cos\theta_H$ (red line), as shown in Figure S4 and the magnetization reversal process of the UMM is accompanied by modified Kondorsky model[1].

ΔR_{xy} of YIG (10 nm)/NiCoO (2.5 nm)/Pt (5.0 nm)

Figure S5 shows ΔR_{xy} of YIG (10 nm)/NiCoO (2.5 nm)/Pt (5.0 nm) as a function of temperature, as defined in the Figure 2a of the main text. Apparently, the results of d=2.5 nm are similar to that of YIG (10 nm)/NiCoO (1.25 nm)/Pt (5.0 nm) in Fig.4 in the main text.

Microstructural and magnetic characterization

The thickness of constituent layers was characterized by X-ray reflectivity (XRR) using a Bruker D8 Discover X-ray diffractometer. Pt and YIG layers are 5±0.4 nm and 10±1 nm in thickness, respectively. Figure S6a shows small angle XRR pattern of NiCoO/Pt heterostructures with NiCoO layer thickness in the region from 0 to 6.0 nm. The 3-dimension atomic force microscopic (AFM) image of 2.5 nm-thick NiCoO layer in Figure S6b indicates the roughness of 0.18 nm. Figure S6c shows transmission electron microscope (TEM) pattern and the selected-area electron diffraction (SAED) of

the 2.5 nm thick NiCoO film.

Anomalous Hall-like effect loops

Figure S7 shows the anomalous Hall-like effect (AHLE) loop of YIG (10.0 nm)/NiCoO (2.5 nm)/Pt (5.0 nm) as a function of temperature. The AHLE is squared at low temperatures and butterfly-shaped at high temperatures. Figure S8 shows AHLE loops for YIG (10.0 nm)/NiCoO (d)/Pt (5.0 nm) with different NiCoO layer thickness, at 65 K. For thick NiCoO layers, the AHLE loop is similar to that of NiCoO/Pt whereas that of thin NiCoO layers is similar to the butterfly shaped loop of YIG/Pt. Apparently, the change from the butterfly shape to the normal one occurs for $1.25<d<2.50$ nm at 65 K.

Supplementary information references

1. Kondorsky, E. On hysteresis in ferromagnetics. *J. Phys. (Moscow)-USSR*, **2**, 161-181 (1940).

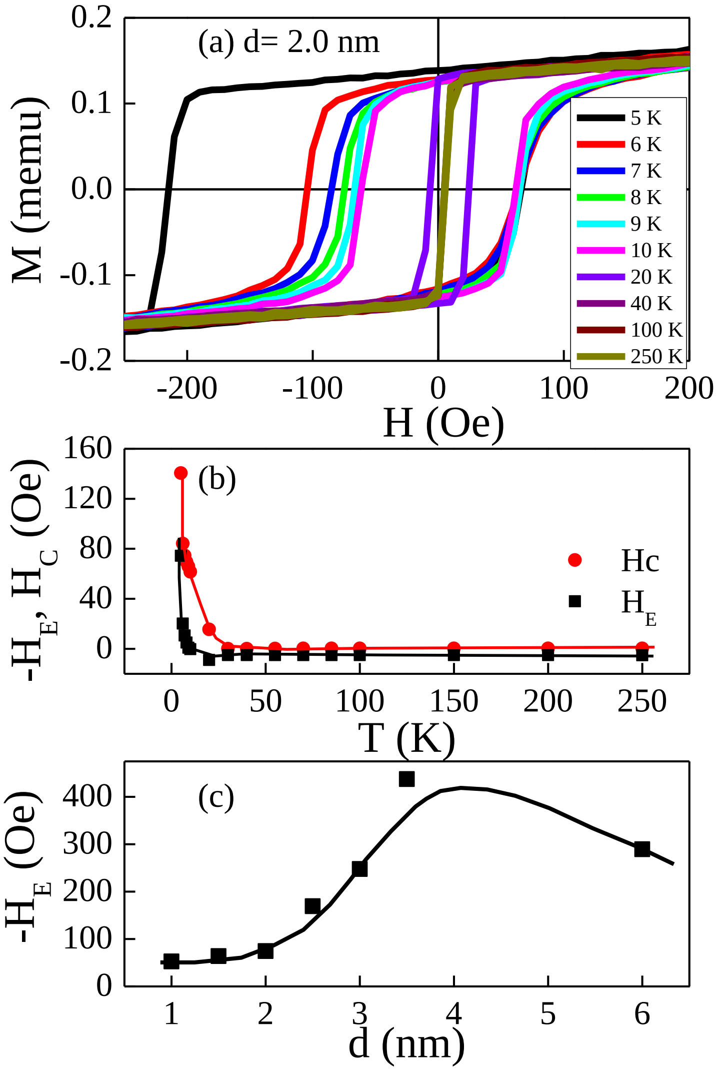

Figure S1 Magnetization hysteresis loops of NiCoO (d)/NiFe (10 nm) bilayers with d=2.0 nm at different temperatures (a). Temperature (b) and NiCoO layer thickness (c) dependencies of exchange bias field H_E (b, c) and H_C (b) of NiCoO/NiFe (10 nm). In (b), d=2.0 nm. In (c), T=5 K.

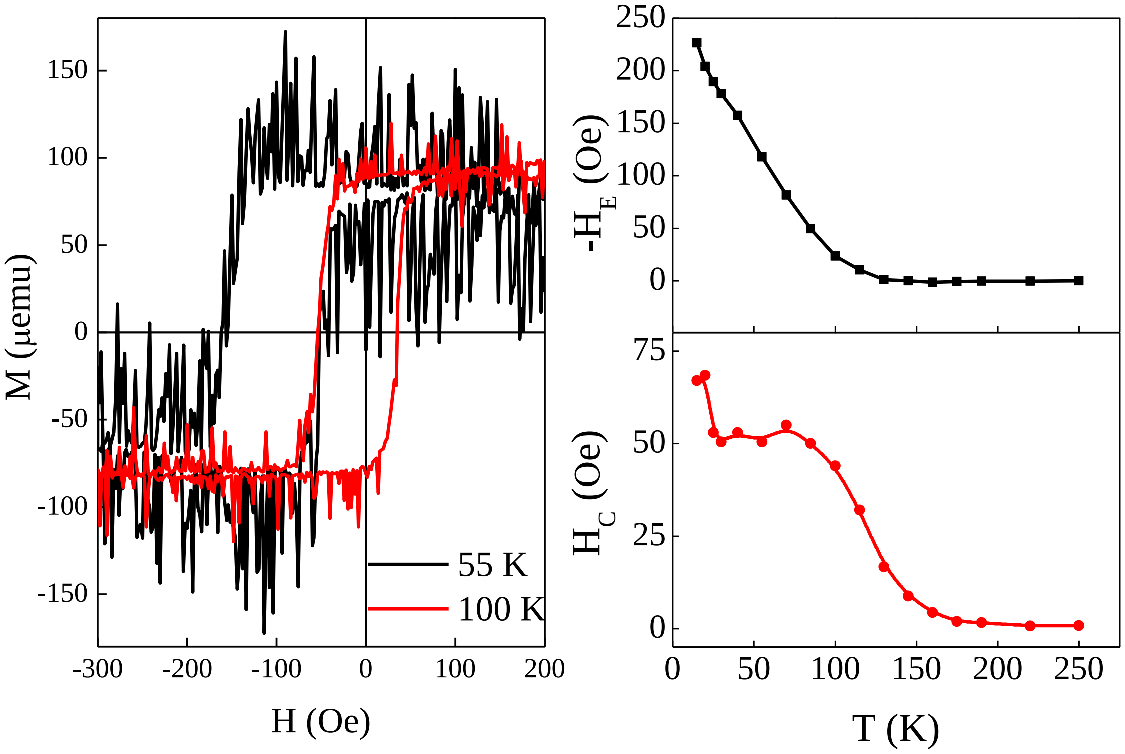

Figure S2 Magnetization hysteresis loops of YIG (10 nm)/ NiCoO (5 nm) bilayers at 55 K and 100 K (a). Temperature dependence of exchange bias field H_E (b) and H_C (c) of YIG (10 nm)/ NiCoO (5 nm).

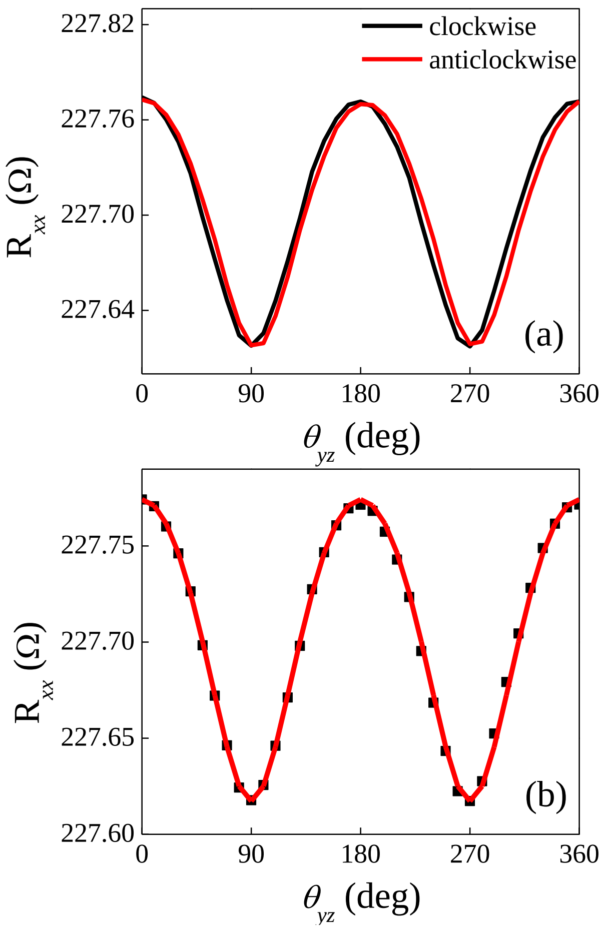

Figure S3 Typical angular dependent SMR results for NiCoO (2.0 nm)/Pt (5.0 nm) with *H*=70 kOe and *T*=5 K.

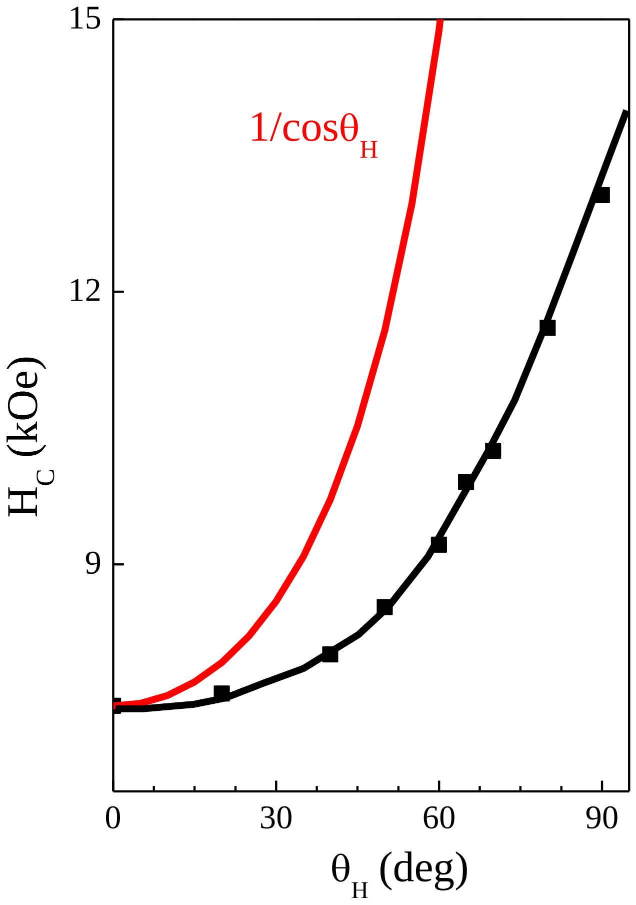

Figure S4 For NiCoO (2.0 nm)/Pt (5.0 nm), out-of-plane coercivity H_C at T=5 K. Black line serves a guide to the eye and red one refers to the depinned motion of domain wall.

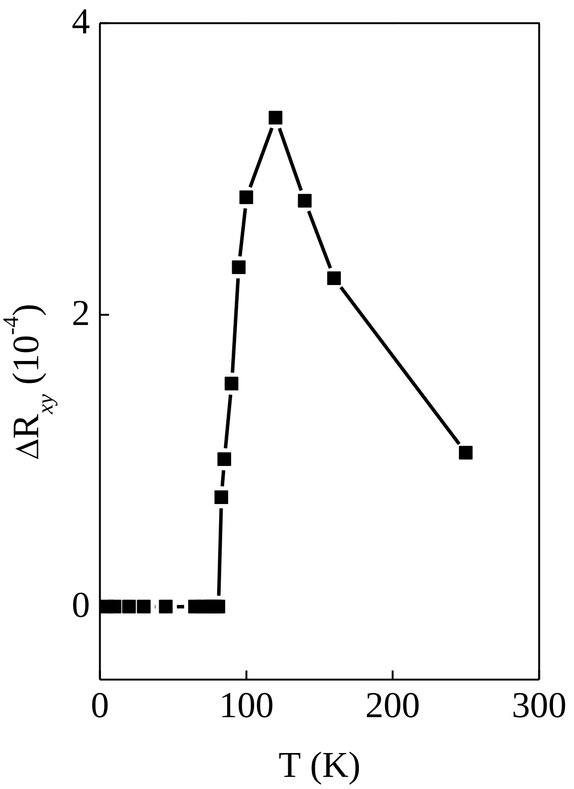

Figure S5 ΔR_{xy} of YIG (10.0 nm)/NiCoO (2.5 nm)/Pt (5.0 nm), as defined in Figure 2a in the main text, as a function of temperature.

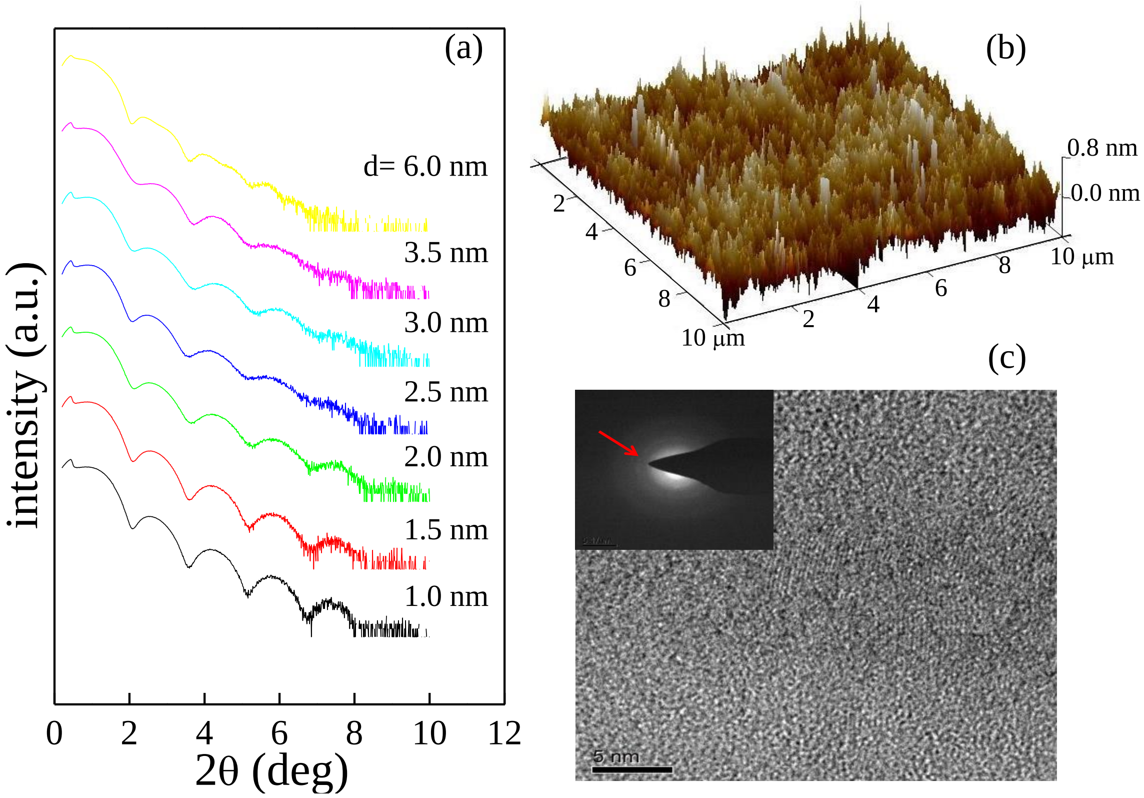

Figure S6 (a) Small angle XRR pattern of NiCoO (d)/Pt samples with *d*=1.0, 1.5, 2.0, 2.5, 3.0, 3.5, and 6.0 (nm). AFM (b) and TEM (c) images of the 2.5 nm thick NiCoO layer on glass substrate. The SAED pattern is shown in the insert of (c).

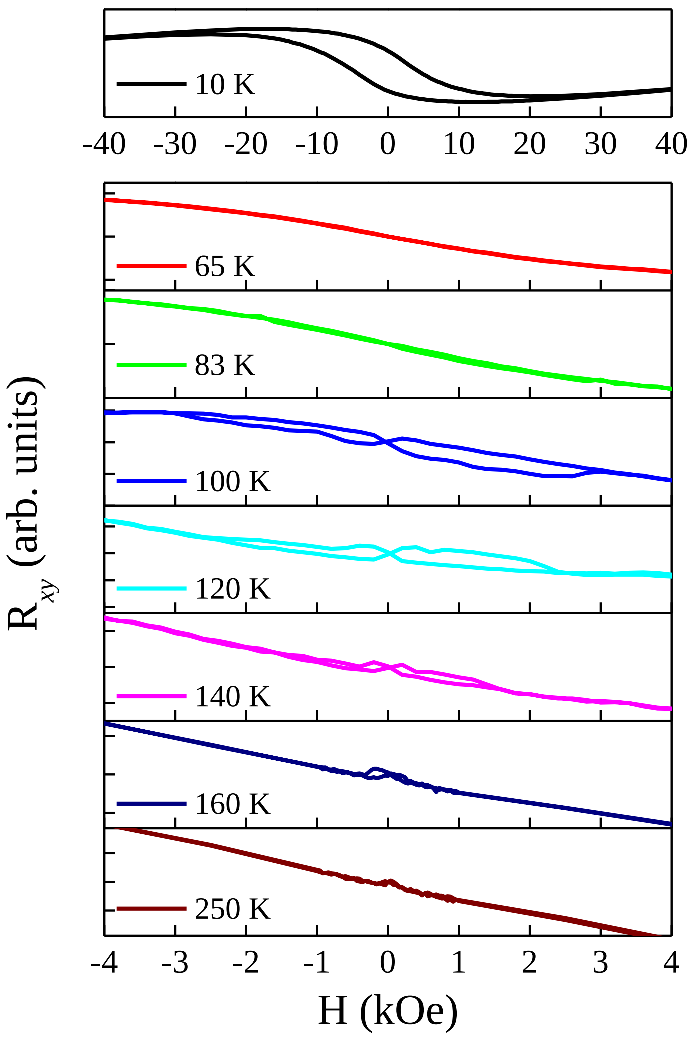

Figure S7 AHLE loops for YIG (10.0 nm)/NiCoO (2.5 nm)/Pt (5.0 nm) as a function of temperature.

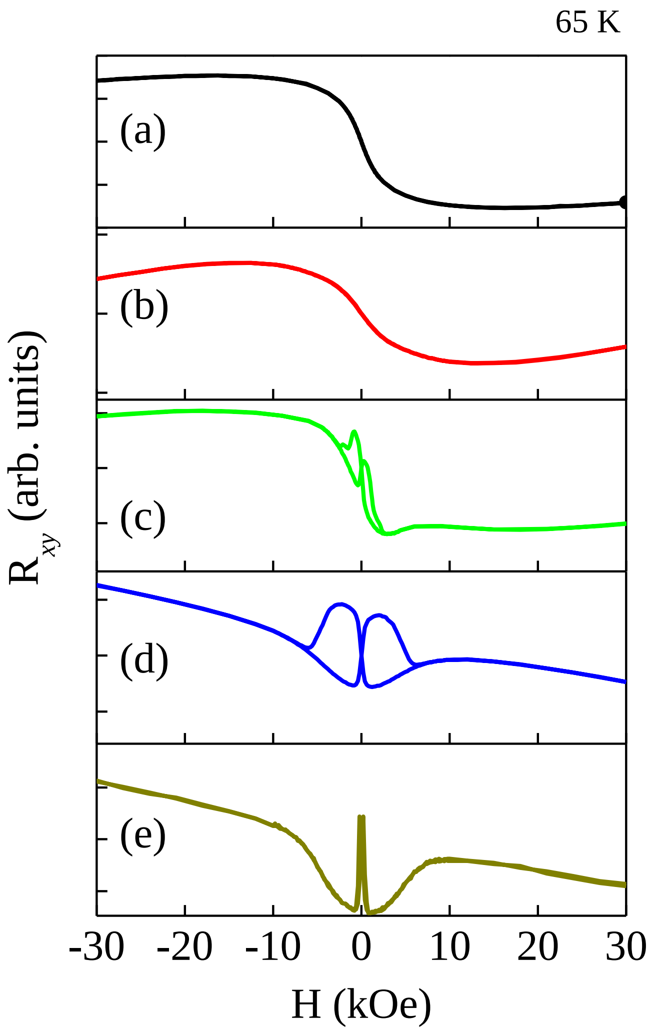

Figure S8 AHLE loops for YIG (10.0 nm)/NiCoO (d)/Pt (5.0 nm) with *d*=3.5(a), 2.50(b), 1.25(c), 0.63(d), and 0(e) (nm) at 65 K.
